# miR-489 induces immunogenic cell death in breast cancer by targeting LAPTM4B

**DOI:** 10.3389/fonc.2026.1832140

**Published:** 2026-05-20

**Authors:** Gourab Gupta, Ryan Titus, Sydney Shaw, Neha Adusumilli, Yogin Patel, Hexin Chen

**Affiliations:** 1 Department of Biological Sciences, University of South Carolina, Columbia, SC, United States; 2 University of South Carolina School of Medicine, Columbia, SC, United States

**Keywords:** breast cancer, ER stress, Immunogenic cell death, microRNA, phagocytosis

## Abstract

**Introduction:**

MicroRNA dysregulation plays a critical role in breast cancer progression, yet the functional significance of many microRNAs in tumor biology and antitumor immunity remains incompletely understood. Here, we investigated the expression pattern, clinical relevance, and biological function of miR-489 in breast cancer.

**Methods:**

miR-489 expression was analyzed in breast cancer tissues, including basal-like/triple-negative breast cancer (TNBC) cohorts, and correlated with patient survival outcomes. Functional assays were performed in TNBC cell lines following miR-489 restoration to assess effects on cell proliferation, colony formation, and endoplasmic reticulum (ER) stress signaling. Markers of immunogenic cell death, including calreticulin exposure, ATP release, and macrophage-mediated phagocytosis, were evaluated. Mechanistic studies identified and validated target genes using molecular and functional rescue approaches.

**Results:**

miR-489 expression was significantly reduced in breast tumors, particularly in the basal-like/TNBC subtype. Higher miR-489 levels were associated with improved patient survival across independent cohorts. Restoration of miR-489 inhibited proliferation and colony formation in TNBC cells and activated ER stress signaling. miR-489 overexpression also induced features of immunogenic cell death, including increased calreticulin surface exposure, ATP release, and enhanced macrophage phagocytosis. Lysosomal-associated protein transmembrane 4 beta (LAPTM4B) was identified as a direct functional target of miR-489. Suppression of LAPTM4B was required for miR-489–mediated ER stress activation and immunogenic cell death-like responses, while LAPTM4B overexpression partially reversed these effects.

**Conclusions:**

Our findings identify a novel miR-489–LAPTM4B regulatory axis that links tumor suppression with immunogenic cell death in breast cancer. This pathway may represent a potential therapeutic target for improving treatment strategies, particularly in TNBC.

## Introduction

Breast cancer remains the most frequently diagnosed malignancy among women worldwide and a leading cause of cancer-related mortality (1, 2). Although substantial therapeutic advances have improved outcomes for patients with hormone receptor–positive and HER2-amplified tumors (3), triple-negative breast cancer (TNBC) continues to pose a major clinical challenge. TNBC, characterized by the absence of estrogen receptor (ER), progesterone receptor (PR), and HER2 expression, accounts for approximately 15–20% of breast cancers and is associated with aggressive clinical behavior, high rates of recurrence, and limited targeted treatment options (4–7). While immune checkpoint blockade has shown promise in a subset of TNBC patients, durable responses remain restricted to a fraction of cases, underscoring the need to better understand tumor-intrinsic mechanisms that influence both tumor survival and antitumor immunity.

MicroRNAs (miRNAs) are small non-coding RNAs that regulate gene expression post-transcriptionally and play fundamental roles in cancer initiation, progression, and therapeutic response. Depending on their targets, miRNAs can function as oncogenes or tumor suppressors. Dysregulation of miRNA expression is a hallmark of many cancers, including breast cancer, where specific miRNAs have been implicated in proliferation, metastasis, stemness, immune evasion, and therapy resistance. Identifying miRNAs that are selectively altered in aggressive subtypes, such as TNBC, may uncover novel therapeutic vulnerabilities and prognostic biomarkers. miR-489 has emerged as a putative tumor-suppressive miRNA in several malignancies (8). Prior studies have suggested that miR-489 regulates cellular proliferation, apoptosis, and autophagy through modulation of oncogenic signaling pathways (9, 10). However, its expression pattern across breast cancer subtypes, prognostic significance, and mechanistic role in TNBC remains incompletely defined. In particular, the influence of miR-489 tumor–immune interactions have not been systematically explored.

Recent advances have highlighted that breast cancer progression is not solely driven by genetic mutations in tumor but is also shaped by dynamic interactions with immune cells within the tumor microenvironment (TME). The increasing evidence indicates that the remodeling of the TME, by infiltration of immunosuppressive immune cells, is associated with unfavorable clinical outcomes, with recent studies identifying novel biomarkers that contribute to these processes (11). Furthermore, emerging large-scale and single-cell analyses have demonstrated that the composition and spatial organization of immune cells within the TME can independently predict clinical outcomes across breast cancer subtypes. Tumor-intrinsic molecular programs, including metabolic reprogramming, have been shown to actively regulate immune signaling pathways, thereby influencing whether tumors adopt immunologically “hot” or “cold” phenotypes that determine responsiveness to immunotherapy (12, 13). In line with this, tumor-suppressive microRNAs such as let-7b-5p have been reported to modulate aerobic glycolysis in breast cancer, highlighting their potential role in reshaping the TME (14). Together, these findings underscore the importance of identifying tumor-intrinsic regulators like microRNAs that link cancer cell–intrinsic signaling to immune activation. In this context, understanding how specific microRNAs influence both tumor biology and immune engagement may reveal novel mechanisms for modulating the tumor immune microenvironment in aggressive subtypes such as TNBC.

Immunogenic cell death (ICD) is a regulated form of cell death that stimulates antitumor immunity through the emission of damage-associated molecular patterns (DAMPs). It is characterized by surface-exposed calreticulin, extracellular ATP release, and activation of endoplasmic reticulum (ER) stress pathways (15–17). ER stress and the unfolded protein response (UPR), mediated through key sensors such as PERK, eIF2α, and IRE1α, are central regulators of ICD (18). Therapeutic strategies capable of inducing ER stress–dependent ICD may convert immunologically “cold” tumors into “hot” tumors, enhancing immune recognition and clearance. However, the endogenous molecular determinants that link tumor suppressor pathways to ER stress–mediated ICD in TNBC are not well understood.

Lysosomal-associated protein transmembrane 4 beta (LAPTM4B) is an oncogenic protein implicated in autophagy regulation, tumor progression, and poor clinical outcomes in multiple cancers, including breast cancer (19). LAPTM4B has been associated with enhanced tumor cell survival and resistance to stress; however, its role in modulating ER stress signaling and immunogenic cell death remains unclear. Notably, bioinformatic predictions and prior expression analyses suggest that LAPTM4B may be a direct target of miR-489 (20), raising the possibility that miR-489 exerts tumor-suppressive and immunomodulatory effects through LAPTM4B suppression.

In this study, we examined the expression, clinical relevance, and functional role of miR-489 in breast cancer. Our results indicate that miR-489 suppresses tumor growth and promotes ICD-associated features by modulating ER stress signaling and LAPTM4B expression in TNBC cells. The identification of the miR-489–LAPTM4B regulatory axis associated with ER stress activation and ICD suggests a potential therapeutic strategy for breast cancer.

## Methods

### Cell line and culture

MDA-MB 231 and HS578T cells were obtained from Dr. Saraswati Sukuma, Johns Hopkins University, in 2008. MDM-MB-231, Hs578T cells and LAPTM4B overexpression in MDA-MB 231 cells (LAPTM4B_OE) were cultured in Dulbecco’s Modified Eagle Medium (DMEM) supplemented with 10% fetal bovine serum, 1% penicillin-streptomycin antibiotic solution, and 1% Sodium Pyruvate. Cells were cultured and maintained at 37 degrees Celsius in a humidified incubator containing 5% CO2. Culture media was replaced every 2 or 3 days, and cells were passaged after reaching approximately 70-80% confluency using trypsinization.

### Transfection

Cells were seeded in appropriate culture vessels (6 well plate or 96 well plate) to a confluency of 60-70% at the time of transfection. Transient transfection of miRNA mimics, inhibitors, or corresponding scramble control was performed using Lipofectamine RNAiMAX (Thermo Fisher Scientific 13778) by briefly diluting miRNA oligonucleotides in Opti-MEM reduced-serum medium (Thermo Fisher Scientific) to a final concentration of 25 nM. In parallel, Lipofectamine RNAiMAX reagent was diluted separately in Opti-MEM as per manufacturer’s instructions and combined with miRNA mixture and incubated for 20 minutes at room temperature to allow formation of lipid–RNA complexes. The resulting transfection mixture was added to respective wells and incubated for 72hrs before harvesting cells for analysis.

### Cell viability assay

To evaluate the effect of miR-489 on cell survival, the indicated cell lines were seeded in 96-well plates in triplicate and transfected with 25 nM scrambled control (scr), miR-489 mimic or miR-489 inhibitor (Inh-489). 72 hours post-transfection, cell viability was assessed using an MTT assay according to the manufacturer’s instructions. Data are presented as mean ± standard error of the mean (SEM) from at least three independent biological replicates (n = 3). Statistical significance was determined using two-way ANOVA followed by appropriate multiple comparisons test.

### Colony formation assay

MDA-MB 231 and HS 578T cells were transfected with scr, miR-489 mimic or miR-489 inhibitor and incubated for 24 hours. Following incubation, cells were collected and seeded for colony formation assays at a density of 4,000 cells per well in 6-well plate. Cells were incubated for one week at 37 C in a humidified atmosphere containing 5% CO_2_ to allow colony formation. Colonies were then washed with 1× phosphate-buffered saline (PBS), fixed with 100% methanol, and stained with 0.5% crystal violet. Visible colonies were photographed.

### Extracellular ATP measurement

Respective cell lines were seeded in a 6 well plate at a density of 3×10^5^ cells/well and transfected with 28nM scr or miR-489 the day after. 72 hours post-transfection, cell culture supernatant was collected and tested for ATP. Extracellular ATP levels were determined using the Luminescent ATP Detection Assay kit (ab113849, Abcam) according to the manufacturer’s instructions. Luminescence intensity was detected using Modulus from Turner BioSystems. Data are presented as mean ± standard error of the mean (SEM) from at least three independent biological replicates (n = 3). Statistical significance was determined using unpaired two-tailed Student’s t test.

### Western blot

Western Blot analysis was performed using standard protocol (21–23). Proteins were isolated after 72 hours of indicated treatment from specified cell lines by using M-PER protein extraction buffer (Thermo Scientific Cat # 78501) added with 10% protease inhibitor, 2% Sodium orthovanadate (1mM), 1% Sodium Fluoride (10mM). p-eIF2α (Cat#5199), p-PERK (Cat#3179), IRE1α (Cat#3294), PERK (Cat#3192), eIF2α (Cat#9722), and GAPDH (Cat#2118) antibodies were purchased from cell signaling technology. LAPTM4B (Cat#18895-1-AP) was purchased from ProteinTech Group.

### Immunofluorescence

Following the indicated treatments, cells were washed with 1× PBS and fixed in 4% paraformaldehyde for 15 minutes. After three washes with 1× PBS, cells were blocked for 1 hour at room temperature in blocking buffer containing 5% BSA. Cells were then incubated overnight at 4 C with Calreticulin primary antibody (GenScript Cat# A02747-50). The following day, cells were washed three times with PBS and incubated with Alexa Fluor 594 goat anti-rabbit IgG (H&L) secondary antibody (Thermo Scientific) for 1 hour at room temperature. After three additional PBS washes, nuclei were counterstained with DAPI for 15 minutes, and coverslips were mounted onto slides. Fluorescence images were acquired using a confocal microscope with Cy3 and DAPI channels.

### Phagocytosis assay

THP-1 cells were seeded in 6-well plates at a density of 1.2 × 10^6^ cells per well and differentiated into macrophage-like cells by treatment with 150 nM phorbol 12-myristate 13-acetate (PMA) for 24 hours. Following the indicated miR-489 treatments, MDA-MB-231 and HS578T cells were harvested using Accutase (Cat#07922) and counted. Tumor cells were then labeled with 5 μM carboxyfluorescein diacetate succinimidyl ester (CFSE; eBioscience) according to the manufacturer’s protocol. Equal numbers of labeled cancer cells were co-cultured with PMA-differentiated THP-1 cells for 24 hours. After co-culture, these cells were collected and stained with PE-conjugated anti-mouse/human CD11b antibody (BioLegend, Cat#101408) for flow cytometric analysis. Phagocytosis was quantified as the percentage of CFSE-positive cells within the CD11b^+^ gated population. Gating strategy for phagocytosis experiments are demonstrated in **Supplementary Figure 3B**. Data are presented as mean ± standard error of the mean (SEM) from three independent biological replicates (n = 3) for MDA-MB-231 cells and two replicates (n = 2) for Hs578T cells. Statistical significance was assessed using an unpaired two-tailed Student’s t-test.

### Flowcytometry

Single-cell suspensions obtained from the indicated cells were quantified using a Bio-Rad^®^ automated cell counter. For each staining condition, 1 × 10^6^ cells were resuspended in 100 µL of washing buffer for immune cell staining. Cells were first incubated with CD16/32 Fc receptor blocking antibody (1:1000 dilution) for 5 minutes to prevent nonspecific binding. Subsequently, cells were stained with the appropriate antibodies for running in the BD Accuri C6 or BD FACSymphony A5 Cell Analyzer. Gating strategies for calreticulin and % of phagocytosis are demonstrated in **Supplementary Figures 3A, B**.

### Statistical analysis

All statistical analyses were performed using GraphPad Prism unless otherwise stated. Data from *in vitro* experiments are presented as mean ± standard error of the mean (SEM) from at least three independent biological replicates (n = 3). Comparisons between two groups were performed using an unpaired two-tailed Student’s t-test. For experiments involving more than two groups, statistical significance was determined using unpaired Student’s t test, one-way or two-way analysis of variance (ANOVA) followed by appropriate multiple comparison tests as indicated in the figure legends. For analysis of publicly available datasets, Statistical analyses were performed using R version 4.5.2. miRNA expression differences between tumor and normal tissues were evaluated using the Wilcoxon rank-sum test. Pairwise comparisons among breast cancer molecular subtypes were performed using the Wilcoxon rank-sum test with continuity correction. Kaplan–Meier survival curves were generated to evaluate the association between miR-489 expression and overall survival in breast cancer cohorts. Survival differences between groups were assessed using the log-rank (Mantel–Cox) test, and hazard ratios (HR) with 95% confidence intervals (CI) were reported where applicable. Correlation analyses between gene expression levels were performed using Spearman’s rank correlation coefficient. A p-value < 0.05 was considered statistically significant. Statistical significance is indicated as follows: *p <0.05,** *p <*0.01,*** *p <*0.001,**** *p <*0.0001.

## Results

### miR-489 is downregulated in breast cancer

**Figure 1:**
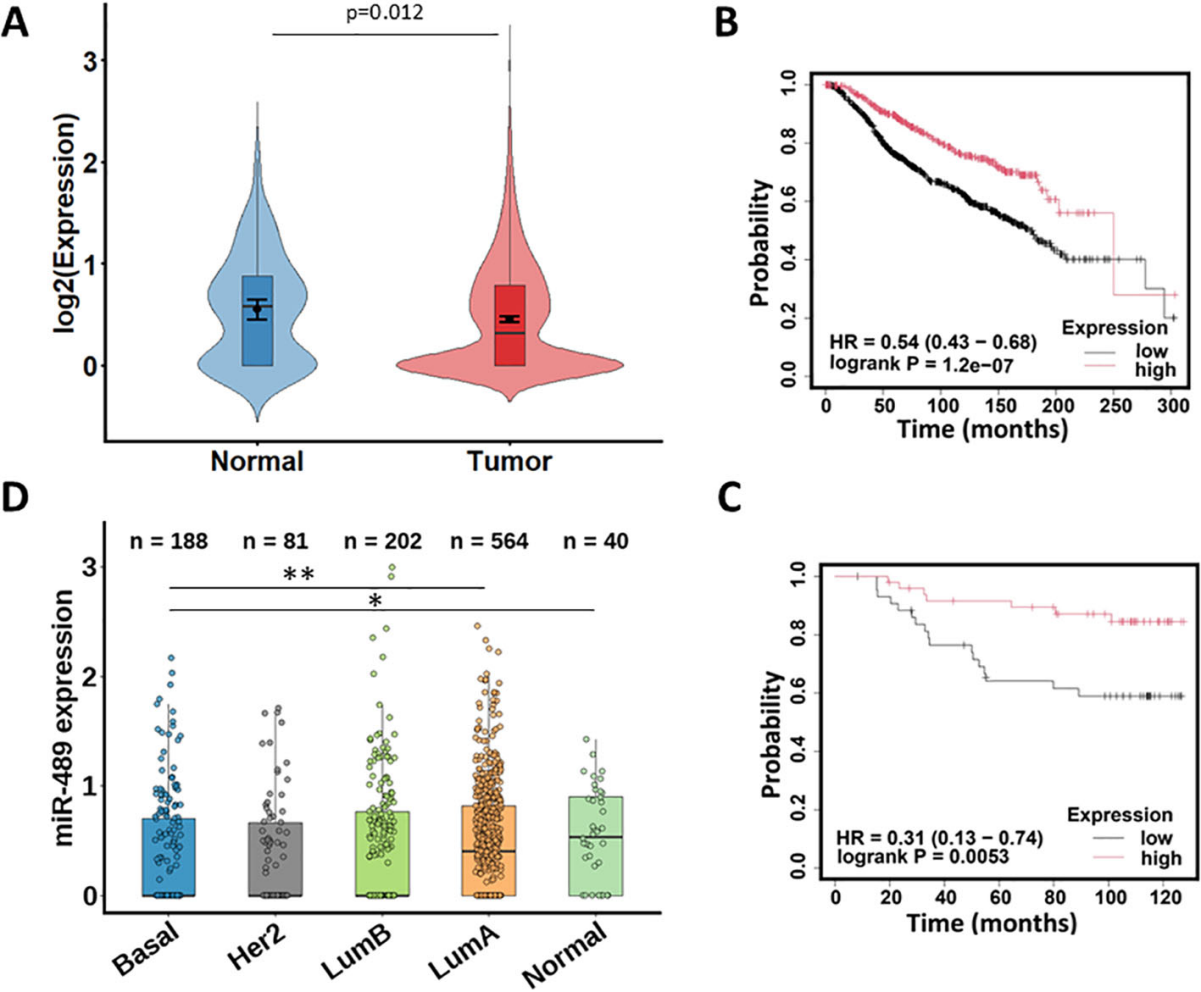
miR-489 expression and survival analysis in breast cancer datasets. **(A)** Analysis of miRNA expression data from the TCGA-BRCA using Wilcoxon rank-sum test. Violin plots showing log2-transformed miR-489 expression levels in TCGA-BRCA primary tumor (n = 1091) and solid tissue normal samples (n = 104) (https://www.cancerimagingarchive.net/collection/tcga-brca/). The center line represents the median; error bars denote mean ± 95% confidence interval. **(B, C)** Kaplan–Meier survival analyses comparing overall survival between patients with high and low miR-489 expression using Kaplan Meier plotter (36). Survival analysis in the METABRIC cohort (37, 38) **(B)** and GSE19783 cohort (39–41) **(C)**. **(D)** miR-489 expression in subtypes of breast cancers from the TCGA-BRCA cohort. Statistical significance was determined by pairwise comparison using Wilcoxon rank sum test with continuity correction. *p<0.05; **p<0.01.

miR-489 is a tumor suppressor microRNA which is downregulated in a few types of cancers. Analysis of miRNA expression data from the TCGA-BRCA cohort demonstrated that miR-489 is significantly downregulated in primary breast tumors compared to normal samples (**Figure 1A**). Kaplan–Meier survival analysis demonstrated that high miR-489 expression was significantly associated with improved overall survival in both the METABRIC and GSE19783 cohorts (**Figures 1B, C**). Analysis of miR-489 expression across molecular subtypes in the TCGA-BRCA cohort revealed subtype-specific differences (**Figure 1D**). miR-489 levels were significantly lower in basal-like tumors compared to other breast cancer subtypes, including HER2-enriched, Luminal B tumors, while normal and luminal subtypes, particularly Luminal A, showed comparatively higher miR-489 expression. These findings indicate that miR-489 is particularly downregulated in basal-like (triple-negative) breast cancer, supporting its potential subtype-specific tumor suppressor role.

### miR-489 overexpression in TNBC cell lines inhibits cell growth and activates ER stress pathways

**Figure 2:**
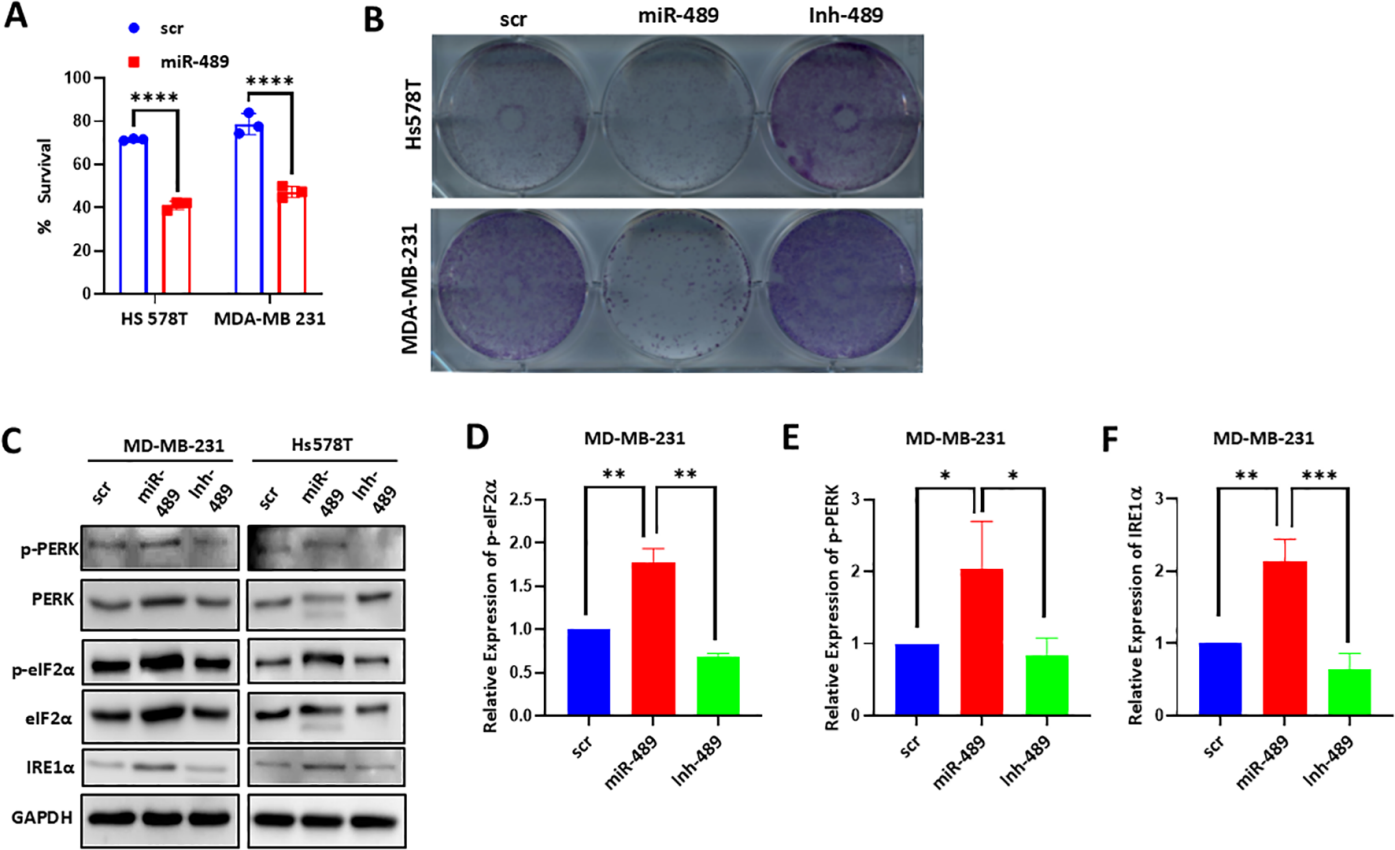
Effect of miR-489 modulation on TNBC cell viability and ER stress signaling. **(A)** Cell viability analysis of MDA-MB-231 and Hs578T cells transfected with scrambled control or miR-489 mimic measured by MTT assay. Data represent mean ± SEM from three independent experiments (n = 3). Statistical significance was determined using two-way ANOVA. ****, p<0.0001. **(B)** Colony formation assay showing the long-term effects of miR-489 mimic or inhibitor on clonogenic survival of TNBC cells. Colonies were stained with crystal violet for visualization. **(C)** Immunoblot analysis of ER stress markers in MDA-MB-231 and Hs578T cells transfected with scrambled control, miR-489 mimic or miR-489 inhibitor. **(D–F)** Quantification of relative expression of phosphorylated eIF2α **(D)** phosphorylated PERK **(E)** and IRE1α **(F)** in MDA-MB-231 cells following miR-489 overexpression or inhibition. Data are presented as mean ± SEM from three independent experiments (n = 3). **(D–F)**. Statistical significance was determined Student’s t-test. Statistical significance between groups is indicated as follows: *p < 0.05, **p < 0.01, ***p < 0.001, ****p < 0.0001.

To examine the effect of miR-489 overexpression on the growth of breast cancer cells, we transiently transfected miR-489 mimic in MDA-MB-231 and Hs578T cells and measured cell growth by MTT assay (**Figure 2A**). Our results clearly indicated that miR-489 overexpression induced significant inhibitory effect on TNBC cell growth. To better understand the role of miR-489 expression on tumorigenicity, MDA-MB-231 and Hs578T cells were transfected with mimic and an inhibitor of miR-489, and seeded at low density to assess their colony formation ability (**Figure 2B**). Our results showed that cells transfected with miR-489 mimic yielded significantly less colonies than the control. Conversely, more colonies were observed in cells transfected with the inhibitor of miR-489. To understand the underlying mechanisms by which miR-489 reduces cell proliferation and colony formation, we considered that miR-489 overexpression has previously been shown to block autophagy at a late stage, which may lead to ER stress. To this end, we examined key markers of the unfolded protein response (UPR). Overexpression of miR-489 in MDA-MB-231 cells resulted in increased expression of ER stress markers, including phosphorylated eIF2α, phosphorylated PERK, and IRE1α indicating activation of the ER stress pathway (**Figures 2C–F**). These findings suggest that miR-489–mediated inhibition of cancer cell growth may be driven by activation of ER stress–dependent death pathways.

### miR-489 overexpression in TNBCs induces hallmarks of ICD

**Figure 3:**
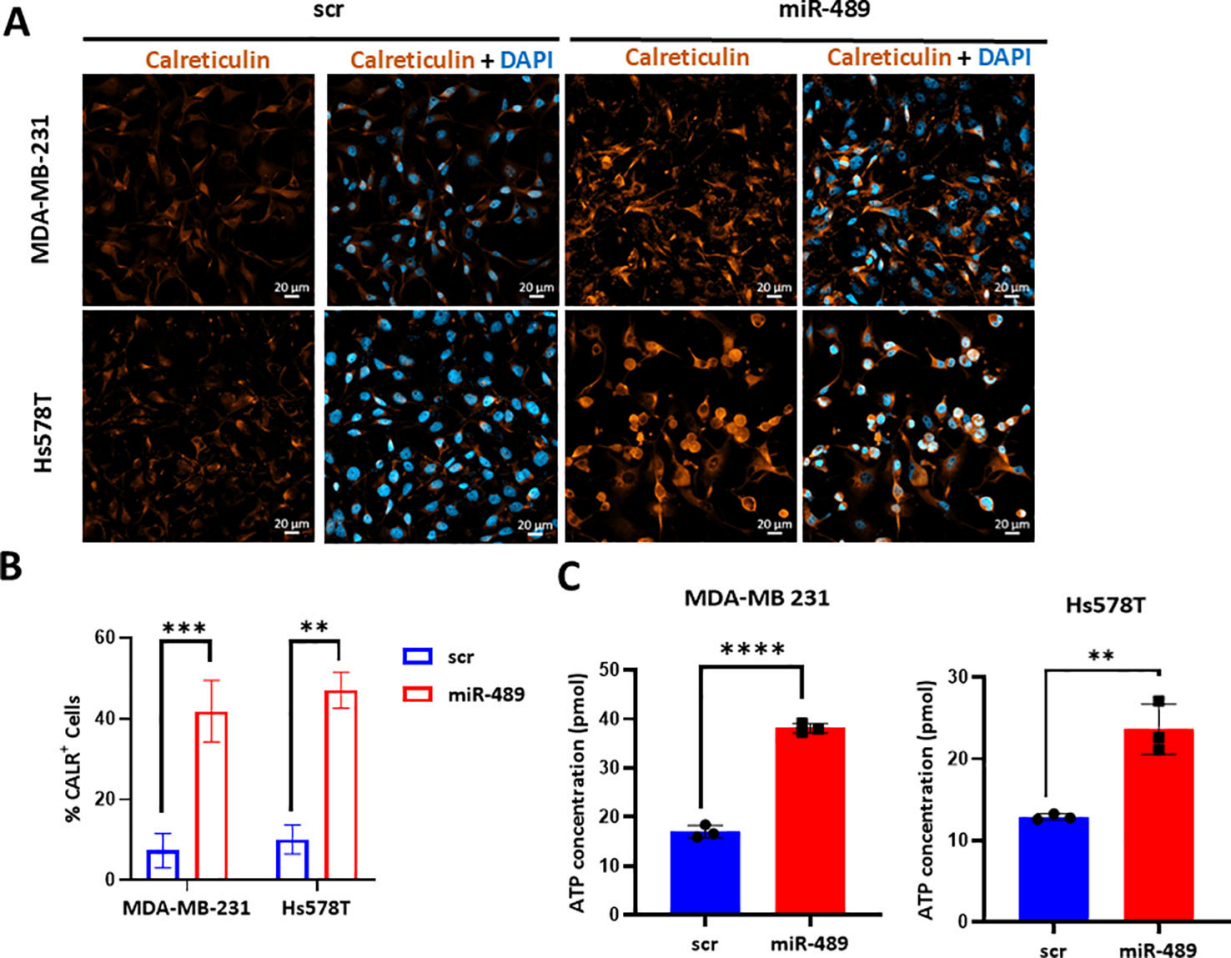
Detection of immunogenic cell death markers following miR-489 overexpression. **(A)** Immunofluorescence staining of calreticulin in MDA-MB-231 and Hs578T cells following transfection with scrambled control or miR-489 mimic. Calreticulin (orange) shown as single channels for each condition along with merged images of Calreticulin and nuclei stain (DAPI, blue). Scale bar, 20 μm. **(B)** Quantitative expression of surface calreticulin levels using Flowcytometry after miR-489 overexpression in TNBC cells. **(C)** Quantification of extracellular ATP levels in culture supernatants of MDA-MB-231 cells and Hs578T cells transfected with scrambled control or miR-489 mimic. **(B, C)** Data are presented as mean ± SEM from three independent experiments (n = 3). Statistical analysis was performed using Student’s t-test. Statistical significance between groups is indicated as follows: *p < 0.05, **p < 0.01, ***p < 0.001, ****p < 0.0001.

Blockade of autophagy can lead to enhanced endoplasmic reticulum (ER) stress, a key cellular stress response known to promote the induction of ICD (24). To determine whether miR-489–induced ER stress leads to ICD, we first investigated the expression of Calreticulin on cell surface. Immunofluorescence analysis revealed enhanced calreticulin expression in miR-489–overexpressing cells compared with scramble controls in both MDA-MB-231 and Hs578T cell lines (**Figure 3A**). We also performed flowcytometric analysis to verify the cell surface expression of calreticulin (**Figure 3B**). Another hallmark of ICD is the release of ATP in extracellular space. We quantified the concentration of ATP in cell culture supernatant with TNBCs transfected with scrambled (scr) RNA and miR-489 mimic. miR-489 mimic treatment significantly increased extracellular ATP in both MDA-MB-231 and Hs578T cell lines (**Figure 3C**). Together, these data support the hypothesis that miR-489 overexpression induces hallmarks of ICD in TNBC cells.

### miR-489 promotes immunogenic phagocytic clearance of tumor cells

**Figure 4:**
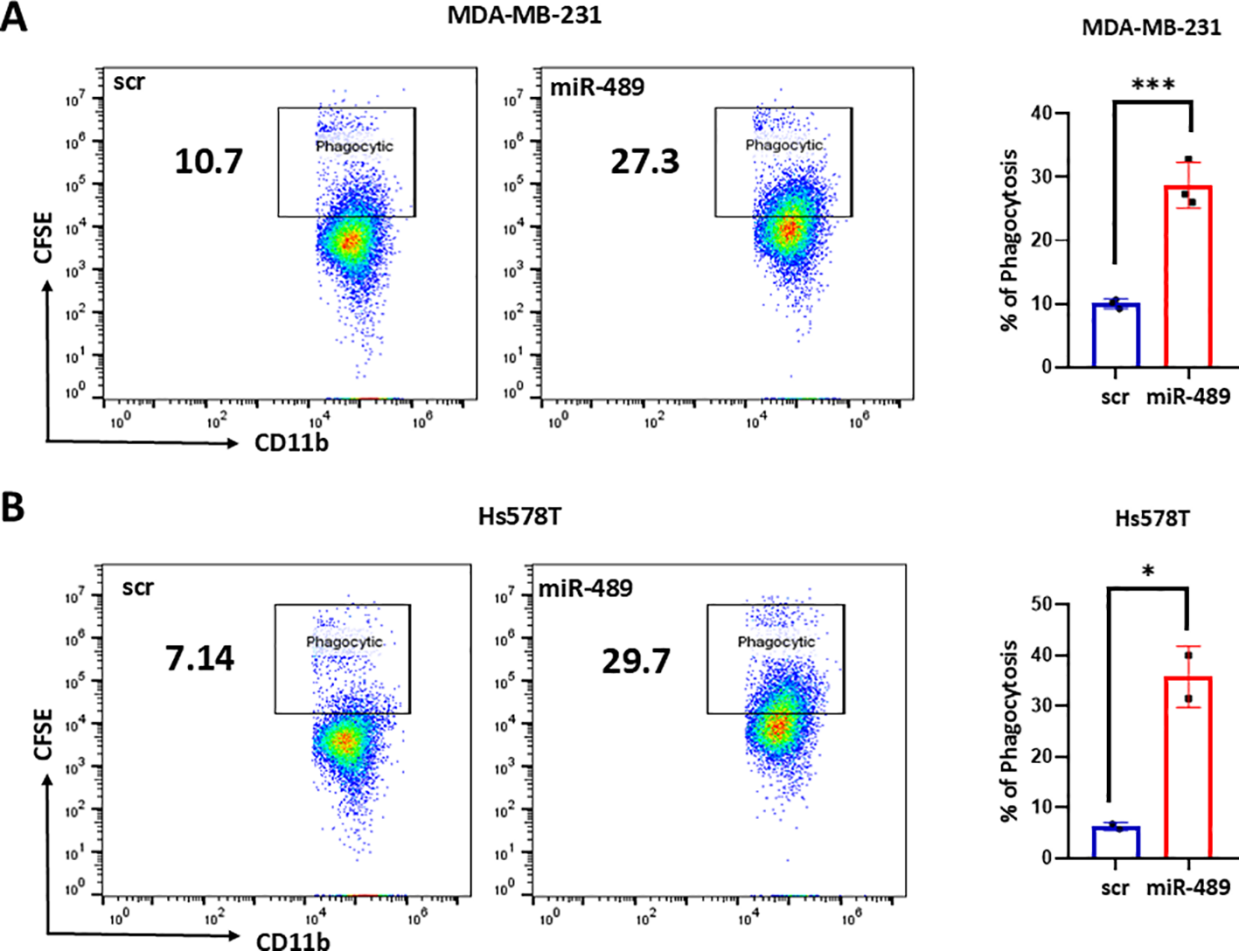
Phagocytosis assay to study immune activation of miR-489. Phagocytosis assay for co-culture of CFSE-labeled TNBC cells with PMA-differentiated THP-1 derived macrophages. Quantification of Phagocytic activity measured by flowcytometry analysis of CFSE-positive MDA-MB-231 cells **(A)** and Hs578T **(B)** cells within the CD11b^+^ macrophage population. Data are presented as mean ± SEM from three independent experiments (n = 3). Statistical analysis was performed using Student’s t-test. Statistical significance between groups is indicated as follows: *p < 0.05, **p < 0.01, ***p < 0.001, ****p < 0.0001.

To further explore if miR-489 induced cell death is immunogenic, we cocultured PMA differentiated THP-1 macrophage-like cells with TNBCs transfected with scramble or miR-489 mimic. The cancer cells were pre-labeled with CFSE, allowing the detection of this dye accumulation in THP-1 derived macrophages after coculture as a result of phagocytosis. THP-1 derived macrophages cocultured with miR-489 overexpressing cells clearly showed an increase in phagocytosis, almost a three-fold increase compared to the control (**Figures 4A, B**). These results indicate that miR-489–induced cell death is not tolerogenic but instead promotes active immune engagement, likely driven by enhanced DAMP signaling associated with ICD.

### miR-489 induces ER stress by targeting LAPTM4B

**Figure 5:**
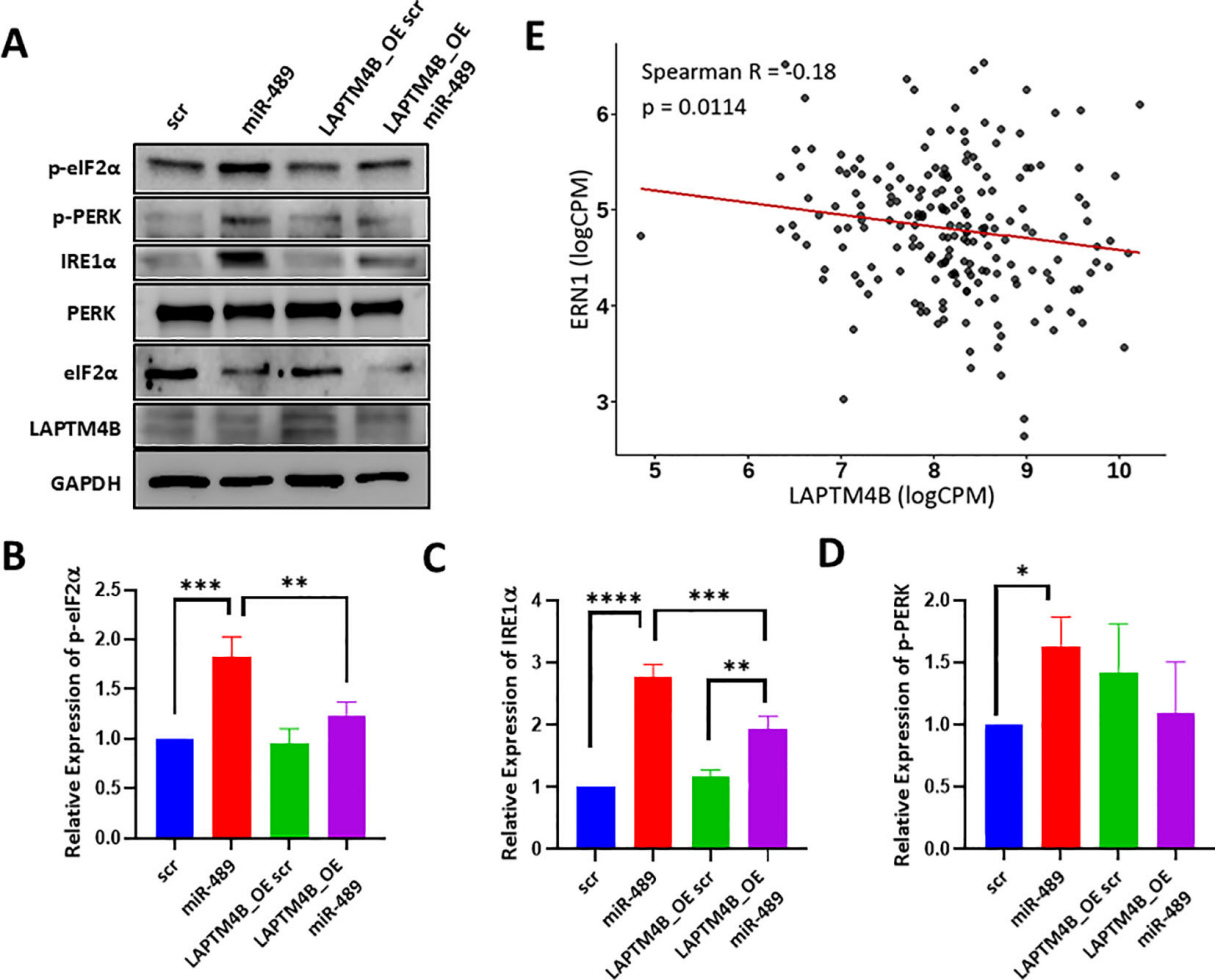
Analysis of ER stress signaling in relation to LAPTM4B expression. **(A)** Western blot analysis of ER stress signaling components in MDA-MB-231 cells transfected with scrambled control or miR-489 mimic in the presence or absence of LAPTM4B overexpression (LAPTM4B_OE). **(B–D)** Quantification of ER stress markers including phosphorylated eIF2α **(B)**, IRE1α **(C)** and phosphorylated PERK **(D)** from immunoblot analyses. Statistical analysis was performed using one-way ANOVA with multiple comparison testing. Correlation was calculated using Spearman’s correlation. Statistical significance between groups is indicated as follows: *p < 0.05, **p < 0.01, ***p < 0.001, ****p < 0.0001. **(E)** Correlation analysis scatter plot showing the relationship between *LAPTM4B* and *ERN1* (IRE1α) mRNA expression levels (logCPM) in basal-like breast cancer samples from the TCGA-BRCA cohort. Each dot represents an individual tumor sample. The red line indicates the fitted regression line. Correlation was assessed using Spearman’s rank correlation coefficient.

To find out a molecular target of miR-489 associated with ER stress response, we looked back at microarray and molecular target prediction algorithms used in our previous publication (20). LAPTM4B emerged as suitable target that has 3’ UTR binding sites for miR-489 and was known to regulate autophagy. This raises the question of whether miR-489 can also mediate ER stress through inhibition of LAPTM4B expression (20). Although miR-489 has a weak but significant correlation with LAPTM4B in TCGA-BRCA cohort (**Supplementary Figure 1**) (25), the effect is more pronounced in the downstream targets of LAPTM4B and its functional phenotypes, one of which is the induction of ER stress. Using MDA-MB-231 cells overexpressing LAPTM4B, we performed Western blot analysis following transient transfection with either scrambled control or miR-489 mimic. The results show activation of ER stress pathways by miR-489, as evidenced by increased phosphorylation of PERK and eIF2α, along with elevated IRE1α protein levels (**Figure 5A**). Total PERK and eIF2α levels remained relatively unchanged, indicating specific activation of the unfolded protein response rather than global alterations in protein expression. These findings confirm that miR-489 induces ER stress signaling through activation of the PERK–eIF2α and IRE1α branches. Importantly, ectopic overexpression of LAPTM4B attenuated miR-489–mediated ER stress activation. In cells co-expressing miR-489 and LAPTM4B, the levels of phosphorylated PERK, phosphorylated eIF2α, and IRE1α were markedly reduced compared to miR-489 expression alone, indicating partial reversal of the ER stress phenotype (**Figures 5B–D**). This rescue effect suggests that LAPTM4B functions downstream of miR-489 and that restoration of LAPTM4B expression counteracts miR-489–induced ER stress signaling, thereby reverting the associated cellular phenotype. IRE1α particularly shows a strong increase in expression upon induction by miR-489 overexpression and the most significant attenuation upon LAPTM4B mediated revival. Because XBP1 is a downstream target of IRE1α, correlation analysis from the TCGA-BRCA dataset exhibited a modest but statistically significant inverse association between LAPTM4B and XBP1 expression (R = −0.10, p = 7.3 × 10^-8^) (**Supplementary Figure 2**). This leads us to believe that miR-489 mediated UPR and ER stress response is particularly accelerated by IRE1α through the downregulation of LAPTM4B. Correlation analysis in the basal-like (predominantly triple-negative) subtype of the TCGA-BRCA cohort revealed a modest but statistically significant inverse association between LAPTM4B and IRE1α expression (Spearman r = −0.18, P=0.0114; **Figure 5E**). Higher LAPTM4B expression was associated with lower IRE1α transcript levels, suggesting a potential negative regulatory relationship between these genes within basal breast tumors.

### miR-489 promotes ICD via LAPTM4B modulation

**Figure 6:**
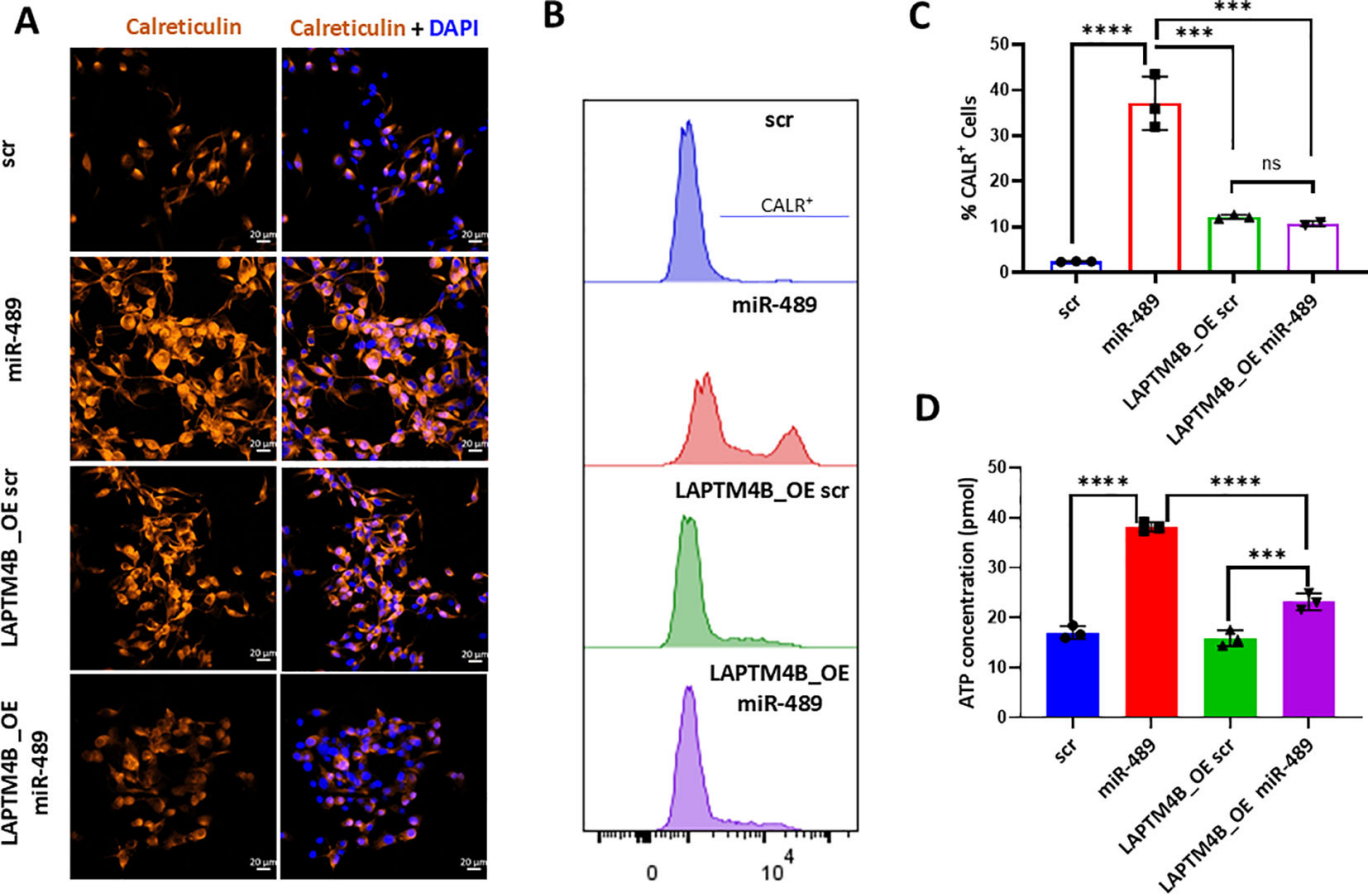
Analysis of immunogenic cell death markers following LAPTM4B modulation. **(A)** Immunofluorescence staining of calreticulin in MDA-MB-231 cells transfected with scrambled control (scr) or miR-489 mimic in the presence or absence of LAPTM4B overexpression (LAPTM4B_OE). Cells were fixed and stained for calreticulin, and nuclei were counterstained with DAPI. Representative confocal images are shown. Calreticulin (orange) shown as single channels for each condition along with merged images of Calreticulin and nuclei stain (DAPI, blue). Scale bars, 20 µm. **(B)** Representative flow cytometry histograms showing cell surface calreticulin expression of MDA-MB-231 cells transfected with scrambled control (scr) or miR-489 mimic in the presence or absence of LAPTM4B overexpression. **(C)** Quantification of calreticulin-positive cells determined by flow cytometry. **(D)** Measurement of extracellular ATP levels in culture supernatants of MDA-MB-231 cells under the indicated conditions. ATP concentrations were determined using a luminescence-based ATP detection assay. **(C, D)**. Statistical analysis was performed using one-way ANOVA followed by multiple comparison testing. Data are presented as mean ± SEM from three independent experiments (n = 3). Statistical significance between groups is indicated as follows: ***p <0.001, ****p <0.0001.

Because LAPTM4B overexpression was able to override miR-489–induced ER stress, we next examined whether this effect also extends to other hallmarks of ICD, thereby determining whether LAPTM4B represents a key target through which miR-489 induces ICD. We found that immunofluorescence staining of Calreticulin on the cell surface showed expected increase with miR-489 overexpression in MDA-MB-231 cells and to some extent in LAPTM4B_OE cells. However, the later showed no such effect after miR-489 overexpression (**Figure 6A**). Flowcytometric analysis of cell surface Calreticulin also shows the same effect (**Figures 6B, C**). There is a complete reversal of this phenotype and goes to show the importance of LAPTM4B in the regulation of Calreticulin shuttling to the extracellular membrane because of miR-489 induced ICD. We also looked at extracellular ATP levels as another hallmark of ICD which consistently showed significant elevation following miR-489 overexpression but LAPTM4B restoration diminished this effect (**Figure 6D**). Collectively, these data indicate that miR-489 promotes ER stress–mediated ICD features, including enhanced calreticulin exposure and ATP release at least in part through suppression of LAPTM4B expression.

## Discussion

In this study, we demonstrate that miR-489 promotes immunogenic cell death in breast cancer, revealing a previously unrecognized mechanism of miR-489 as a tumor suppressor microRNA. While miR-489 has previously been reported to function as a tumor suppressor in breast cancer by inhibiting proliferation (9, 10, 26–28), epithelial mesenchymal transition(EMT) (29), autophagy (20) and cancer stem cells (27), our findings extend its role to modulate the immunogenicity of tumor cell death.

Mechanistically, our findings suggest that miR-489 induces features of immunogenic cell death (ICD) by directly targeting LAPTM4B. LAPTM4B is known to regulate lysosomal trafficking, promote autophagy, and cellular adaptation to stress (30). Our previous studies demonstrated that miR-489 overexpression inhibits autophagy by targeting LAPTM4B, thereby sensitizing breast cancer cells to stress signaling–induced cell death (20). Western blot analysis confirmed that miR-489 overexpression activates ER stress signaling, as evidenced by PERK pathway activation, increased IRE1 expression, and enhanced phosphorylation of eIF2α. ER stress signaling can directly promote or sensitize cells to death. Because premature autophagy signaling and endoplasmic reticulum (ER) stress is typically required to induce immunogenic cell death (16, 31, 32), we further investigated this possibility. Consistent with this mechanism, miR-489–overexpressing breast cancer cells exhibited key features of ICD, including the release or surface exposure of damage-associated molecular patterns (DAMPs) such as calreticulin and extracellular ATP. These immunostimulatory signals are known to promote dendritic cell recruitment and activation, facilitating tumor antigen presentation and subsequent phagocytosis by antigen-presenting cells. Consistently, *in vitro* phagocytosis assays confirmed that miR-489 overexpression enhances macrophage-mediated engulfment of tumor cells. Our findings suggest that miR-489-mediated suppression of autophagy represents an alternative strategy for inducing certain hallmarks of immunogenic cell death. This mechanism expands the current understanding of how microRNAs regulate ICD, as many microRNAs may promote immunogenic cell death indirectly through modulation of autophagy pathways.

The identification of the miR-489–LAPTM4B regulatory axis has important therapeutic implications. Our previous work demonstrated that miR-489 overexpression sensitizes tumor cells to chemotherapy by regulating autophagy (20). Autophagy plays a complex, context-dependent role in cancer, often functioning as a cytoprotective mechanism that allows tumor cells to survive nutrient deprivation, hypoxia, and treatment-induced stress (33). Our previous findings suggest that miR-489 overcomes chemoresistance by suppressing LAPTM4B-mediated autophagy (20). In the current study, miR-489 overexpression further redirects the cellular response from stress adaptation to ICD, raising the possibility that miR-489 mimics could synergize with immunotherapy by enhancing tumor antigenicity and stimulating immune activation, thereby potentially improving breast cancer immunotherapy outcomes.

Although our *in vitro* results demonstrate that miR-489 promotes immunogenic cell death in breast cancer cells, highlighting its potential for the development of novel therapeutic strategies, further studies using immunocompetent *in vivo* models are necessary to determine how miR-489 shapes the tumor immune microenvironment. However, the three-base difference between the mouse and human miR-489 seed sequences poses a challenge for directly assessing the function of human miR-489 in immunocompetent mouse models. In addition, LAPTM4B is unlikely to be the only target through which miR-489 regulates tumor cell death, as microRNAs typically modulate multiple genes within interconnected signaling networks. Identifying additional miR-489 targets will provide a more comprehensive understanding of its role in tumor biology. A further limitation of this study is the use of a limited number of TNBC cell lines, as the functional analyses were conducted in only two models (MDA-MB-231 and Hs578T), which may restrict the generalizability of the findings across the heterogeneous TNBC landscape. Moreover, the rescue-oriented experiments examining LAPTM4B function were performed exclusively in MDA-MB-231 cells, primarily due to the availability of a stable LAPTM4B overexpression model in this background. Although the effects of miR-489 on ER stress activation and immunogenic cell death were consistently observed in both TNBC cell lines, restricting the mechanistic rescue analyses to a single model represents an additional limitation and may affect the broader applicability of the conclusions.

The potential relevance of our findings to cancer immunotherapy remains to be determined and represents a possible future direction. While miR-489-mediated induction of ICD features suggests a possible role in enhancing tumor immunogenicity, its impact on immunotherapy responsiveness has not been directly evaluated. Given that strategies promoting ICD may improve responses to immune checkpoint blockade (34, 35), it will be important to determine whether miR-489 restoration can modulate antitumor immune responses in immunocompetent models. Future studies integrating miR-489 modulation with established immunotherapeutic approaches will be necessary to assess its potential to enhance treatment efficacy in breast cancer.

In conclusion, our study identifies a previously unrecognized role for miR-489 in promoting hallmarks of immunogenic cell death through targeting LAPTM4B in breast cancer. These findings reveal an important connection between microRNA-mediated regulation of autophagy and tumor immunogenicity and suggest that modulation of the miR-489–LAPTM4B pathway may represent a promising strategy for enhancing both tumor cell killing and anti-tumor immune responses.

## Data Availability

The original contributions presented in the study are included in the article/supplementary material. Further inquiries can be directed to the corresponding author.
