# Supplementary material for: miR-489 induces immunogenic cell death in breast cancer by targeting LAPTM4B
Source: Front Oncol. 2026 May 20;16:1832140. doi: 10.3389/fonc.2026.1832140 (PMC13230039; doi:10.3389/fonc.2026.1832140)
Supplement: Supplementary file 1 [file DataSheet1.pdf]

Supplementary Data

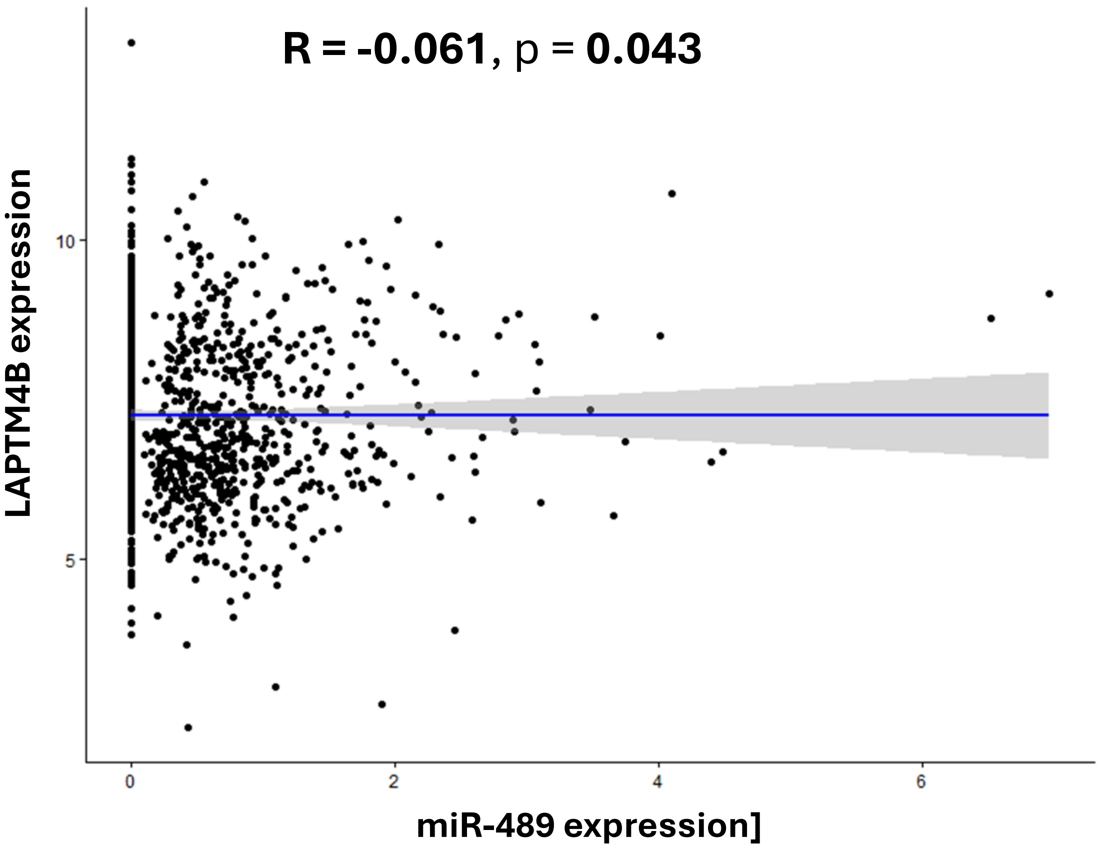

Supplementary Figure 1. Correlation Scatter plot showing the relationship between miR-489-3p and LAPTM4B expression in 1085 breast cancer (BRCA) samples from the ENCORI database[1]. Expression levels are presented as log2(RPM + 0.01) for miR-489-3p and log2(FPKM + 0.01) for LAPTM4B. The blueline represents the linear regression fit. Correlation was assessed using Kendall Rank correlation (R = −0.061, p =0.043).

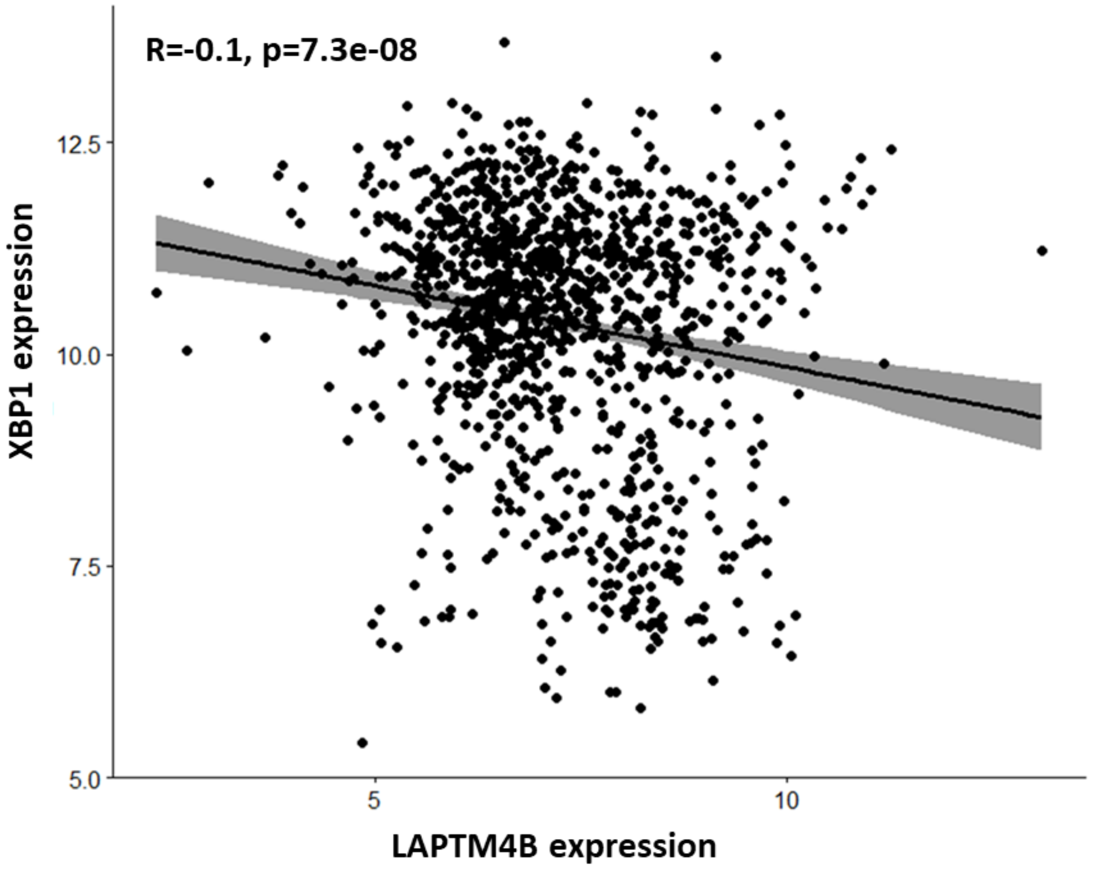

Supplementary Figure 2. Scatter plot showing the relationship between LAPTM4B expression and the ER stress–related gene XBP1 across breast cancer samples (TCGA-BRCA). Each point represents an individual tumor sample. The solid line represents the linear regression fit with the shaded area indicating the confidence interval. Correlation was assessed using Kendall Rank correlation ($R = -0.10$, $p = 7.3 \times 10^{-8}$).

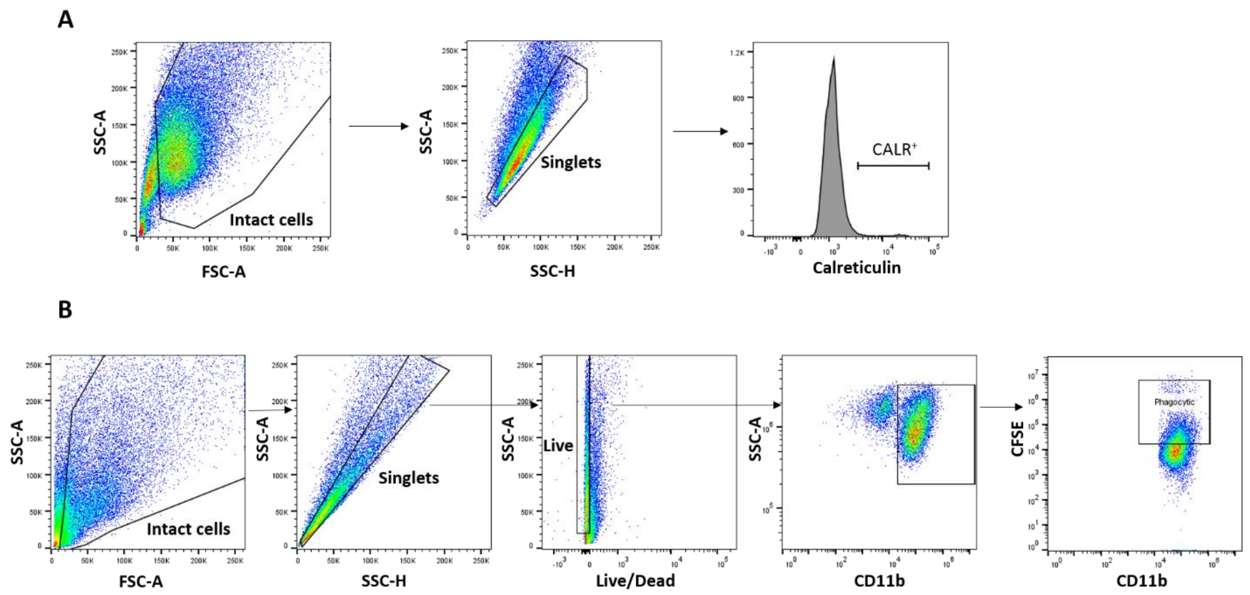

Supplementary Figure 3. Gating strategy for Calreticulin and Phagocytosis experiments

(A) Gating strategy for Calreticulin surface exposure experiment performed by flowcytometry.

(B) Gating strategy for Phagocytosis experiments where % of phagocytosis was determined by CFSE⁺ cells in CD11b+ gated PMA differentiated THP-1 derived macrophages.

Supplementary References

1. Li, J.H., Liu, S., Zhou, H., Qu, L.H. & Yang, J.H. starBase v2.0: decoding miRNA-ceRNA, miRNA-ncRNA and protein-RNA interaction networks from large-scale CLIP-Seq data. *Nucleic Acids Res* **42**, D92-97 (2014).
